# Complete mitochondrial genome of *Baylisascaris* sp. from a striped skunk (*Mephitis mephitis*) in Kansas

**DOI:** 10.1007/s00436-026-08732-z

**Published:** 2026-07-21

**Authors:** Theresa A. Quintana, Lauren E. Camp, Steven A. Nadler, Jeba R.J. Jesudoss Chelladurai

**Affiliations:** 1 Department of Pathobiology, College of Veterinary Medicine, Auburn University, Auburn, AL USA; 2 Veterinary Sciences Department, University of Wyoming, Laramie, WY USA; 3 Wyoming State Veterinary Laboratory, Laramie, WY USA; 4 Department of Entomology and Nematology, University of California, Davis, CA USA

**Keywords:** Mitochondrial genome, Ascaridoidea, Classification, Public health, Animals

## Abstract

*Baylisascaris* species parasitizing skunks have received limited attention compared to the well-characterized raccoon roundworm (*B. procyonis*). We present the first complete mitochondrial genome of a *Baylisascaris* sp. collected in Kansas, United States, from a striped skunk (*Mephitis mephitis*). We morphologically identified the nematode as *Baylisascaris* sp., extracted DNA, sequenced it on the Illumina platform, and *de novo* assembled the complete mitochondrial genome. The complete mitochondrial genome was 14,752 bp, consisting of 12 protein-coding genes, 2 rRNAs, and 22 tRNAs. The genome had a significant AT-skew (70.5%). The mitochondrial genome obtained in this study was 98% identical to that of *B. procyonis* and grouped most closely to *B. procyonis* with 100% bootstrap support in a phylogenetic analysis of ascarid nematodes. The mitochondrial genome generated in this study (GenBank accession no. PZ025724) will benefit future studies in diagnostic epidemiology and systematics and has implications for public health.

**Supplementary Information:**

The online version contains supplementary material available at https://doi.org/10.1007/s00436-026-08732-z.

## Introduction

*Baylisascaris* (Ascarididae, Ascaridinae) is a genus of parasitic nematodes that infects a diverse range of carnivorous and omnivorous mammals, including raccoons, skunks, bears, red pandas, badgers, and mustelids (Sprent 1968; Sapp et al. 2017). The most significant species from a public health perspective is *B. procyonis*, the raccoon roundworm, which can cause neural larva migrans in humans, especially in young children who may inadvertently ingest embryonated eggs from contaminated soil (Kazacos 2001; Graeff-Teixeira et al. 2016).

Historically, *Baylisascaris* nematodes from raccoons were referred to as *B. procyonis* (Stefanski and Zarnowski, 1951) and those from skunks as *B. columnaris* (Leidy, 1856) (Sprent 1968). However, the designation of these nematodes from raccoons and skunks as separate species has been increasingly questioned (Franssen et al. 2013; Choi et al. 2017; Camp et al. 2018; Gu et al. 2024). Morphologically, the two species are almost indistinguishable except for the shape of the denticles (Sprent 1968). Multiple molecular studies have revealed remarkably low nucleotide divergence between raccoon- and skunk-derived worms across mitochondrial markers including cytochrome c oxidase 1 (*cox1*) and 2 (*cox2*) (Franssen et al. 2013; Choi et al. 2017; Camp et al. 2018; Gu et al. 2024), NADH dehydrogenase subunit 2 (*nad2*) (Choi et al. 2017), several transfer RNA (tRNA) genes (Choi et al. 2017), and 12 S ribosomal DNA (Camp et al. 2018). Nuclear DNA genes show the same pattern of low nucleotide divergence, including internal transcribed spacer 1 (ITS-1) and 2 (ITS-2) (Franssen et al. 2013; Camp et al. 2018; Gu et al. 2024), 28S ribosomal DNA, major sperm protein (*msp*), and two exon-primed, intron-crossing loci genes, alcohol/ribitol dehydrogenase (*ard-1*) and histidyl tRNA synthetase (*hars-1*) (Camp et al. 2018). Phylogenetic analyses using single genes, multiple genes, and population genetic models have yielded inconsistent results regarding species boundaries (Franssen et al. 2013; Camp et al. 2018; Gu et al. 2024). Consequently, the current classification of *Baylisascaris* species needs additional investigation in light of available molecular evidence.

Mitochondrial genome studies of *Baylisascaris* species derived from skunks are of particular significance due to the limited data available, with no mitochondrial genome currently reported. Complete mitochondrial genomes have the potential for providing greater resolving power than single-gene analyses (Havird and Santos 2014; Zhang et al. 2021). In this study, we sequenced the complete mitochondrial genome of a skunk-derived *Baylisascaris* sp. on the Illumina platform (GenBank accession no. PZ025724) and examined its phylogenetic relationship with selected species from the family Ascarididae.

## Materials and methods

An immature worm was collected during necropsy from the duodenum of a male striped skunk (*Mephitis mephitis*) from Kansas, that was presented to the Kansas State Veterinary Diagnostic Lab (KSVDL) in 2024 and was transferred to the Parasitology laboratory for identification. It was preserved in 70% ethanol and stored at -20°C until further processing. Total DNA was extracted using the Qiagen DNeasy Blood and Tissue Kit (Qiagen, Valencia, CA) according to the manufacturer’s protocol and eluted in nuclease-free water and stored at − 20°C. Total DNA was quantified using a Qubit™ dsDNA HS Assay Kit (Invitrogen, Eugene, OR). DNA was submitted to CD Genomics (Shirley, NY) for sequencing on an Illumina NovaSeq 6000 platform. Raw sequencing data were obtained in FASTQ format and are available through the National Center for Biotechnology Information (NCBI) sequence read archive (SRA) database (accession number SAMN61218381; BioProject: PRJNA1484093). Reads were trimmed using Trim Galore! version 0.6.10 (de Sena Brandine and Smith 2021) on Galaxy servers (Krueger et al. 2023) to remove adapters and low-quality sequences. Both raw and trimmed reads underwent quality control using Falco version 1.2.4 (Dierckxsens et al. 2016). The trimmed reads were then assembled into a circularized mitochondrial genome using NOVOPlasty version 4.3.5 (Capella-Gutiérrez et al. 2009) on the High-Performance Computing (HPC) system at the Alabama Supercomputer Authority. The assembled genome was annotated with MITOS2 version 2.1.9 to identify protein-coding genes, rRNAs, and tRNAs. tRNA gene annotations were verified using ARAGORN version 1.2.41 (Laslett and Canback 2004). A circularized genome map was created using Proksee version 2026.02.09 (Grant et al. 2023). Genes were concatenated in R version 4.4.2 using the *phylotools* library version 0.2.5 (Zhang 2024).

To perform a phylogenetic analysis, we obtained 11 complete mitochondrial genomes from GenBank for species belonging to the family Ascarididae, with *Ancylostoma caninum* (accession no. NC_012309) used as an outgroup. The final dataset consisted of twelve protein-coding and two rRNA gene sequences for each mitochondrial genome. Sequences were aligned using MAFFT version 7.487 (Katoh et al. 2002), trimmed with trimAI version 1.2 (Nguyen et al. 2014), and concatenated in their genomic order beginning with *nad4L* with custom R scripts run in R version 4.4.2. A maximum-likelihood phylogenetic tree was constructed from the concatenated dataset in IQTREE version 1.6.12 (Trifinopoulos et al. 2016) using partition-specific models (Kalyaanamoorthy et al. 2017) selected by ModelFinder based on Bayesian information criteria (BIC) (Letunic and Bork 2021). The results were visualized with iTOL version 7.3 (Letunic and Bork 2021).

## Results and discussion

**Fig. 1:**
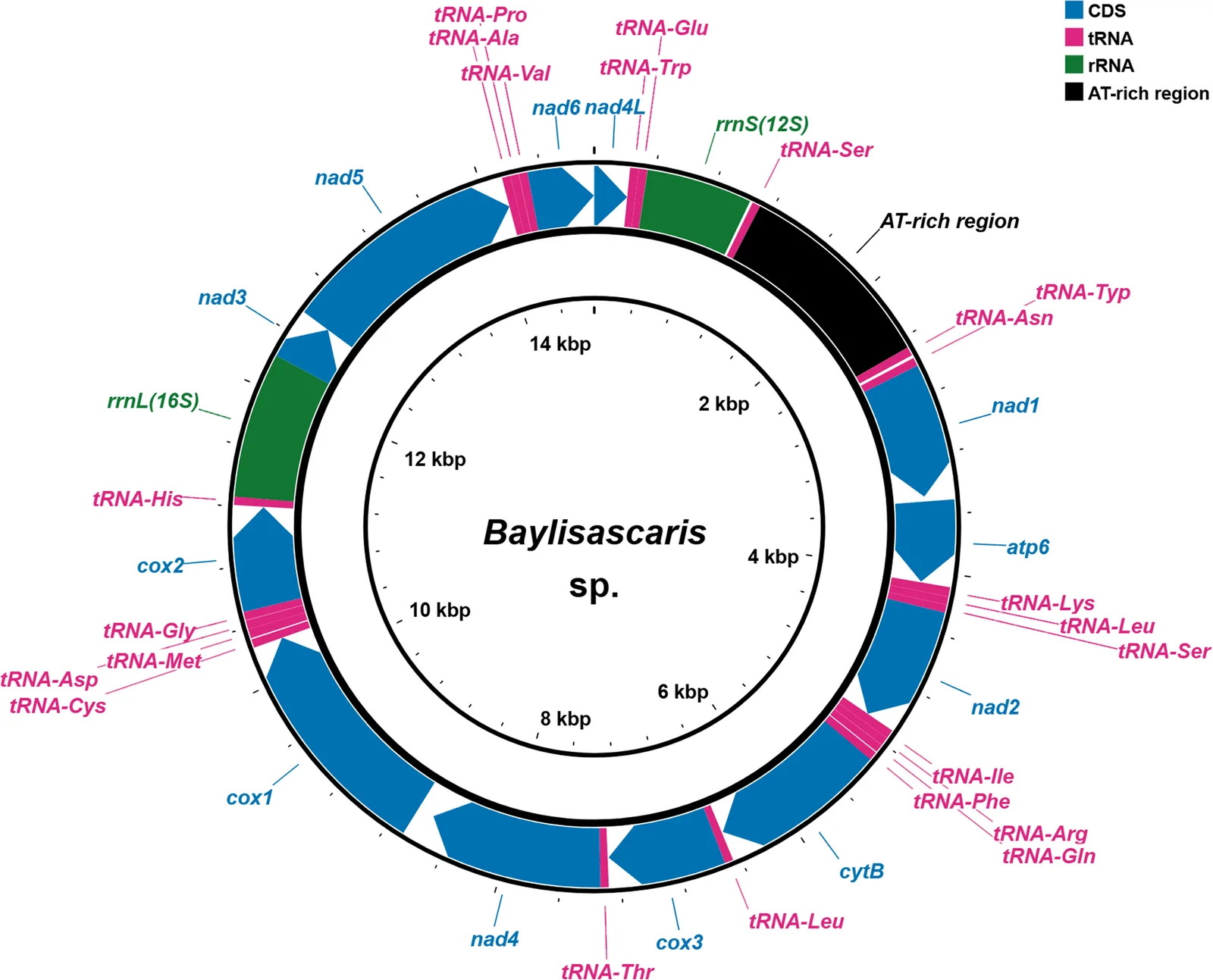
The circularized map of the mitochondrial genome of *Baylisascaris* sp. from a skunk. Gene map showing 12 protein-coding genes (blue), 22 transfer RNAs (tRNAs; pink), 2 ribosomal RNAs (rRNAs; green), and the AT-rich region (black)

We present the first complete mitochondrial genome of *Baylisascaris* derived from a skunk. We used microscopy to examine the immature worm recovered from a striped skunk and confirmed its identification as *Baylisascaris* sp. (Supplementary Fig. 1). The complete mitochondrial genome is 14,752 bp and includes 12 protein-coding genes, 22 transfer RNAs (tRNAs), and 2 ribosomal RNAs (rRNAs). The *atp8* gene is missing, consistent with other members of the family Ascarididae (Jex et al. 2008; Xie et al. 2011). The genome from this study was 98.07% identical to the reference *B. procyonis* complete mitochondrial genome (NC_016200). Figure 1; Table 1 show the gene organization and annotation of the complete mitochondrial genome of the skunk-derived *Baylisascaris*. This skunk *Baylisascaris* uses the invertebrate mitochondrial code (translation code 5). Of the 12 protein-coding genes, 2 start with the codon ATT (*nad4l* and *nad5*), 1 with ATA (*atp6*), 3 with GTG (*nad2*, *cytB*, and *cox3*), and 6 with TTG (*nad1*, *nad4*, *cox1*, *cox2*, *nad3*, and *nad6*). The stop codons include 4 genes ending in TAA (*nad4l*, *nad2*, *nad3*, and *nad5*) and 8 in TAG (*nad1*, *atp6*, *cytB*, *cox3*, *nad4*, *cox1*, *cox2*, and *nad6*). The lengths of the small and large rRNA are 699 and 963 bp, respectively. The nucleotide composition of the genome is approximately 21.9% adenine (A), 8% cytosine (C), 48.6% thymine (T), and 21.5% guanine (G), with a total AT content of approximately 70.5%. Start and stop codon usage, AT content, and genome size are consistent with other mitochondrial genomes of species in the family Ascarididae (Okimoto et al. 1992; Jex et al. 2008; Xie et al. 2011).

**Table 1:** Gene annotation of the mitochondrial genome of *Baylisascaris* sp. from a skunk

Gene name	Gene symbol	Start	End	Size (bp)	Anticodon	Strand	Gene product
NADH dehydrogenase subunit 4 L	*nad4l*	ATT	TAA	234		+	protein
tRNA-Trp	*W*			57	TCA	+	trna
tRNA-Glu	*E*			57	TTC	+	trna
Small ribosomal RNA (12 S rRNA)	*rrnS*			699		+	ribosome, small
tRNA-Ser (UCN)	*S2*			54	TGA	+	trna
tRNA-Asn	*N*			58	GTT	+	trna
tRNA-Tyr	*Y*			56	GTA	+	trna
NADH dehydrogenase subunit 1	*nad1*	TTG	TAG	876		+	protein
ATP synthase F0 subunit 6	*atp6*	ATA	TAG	600		+	protein
tRNA-Lys	*K*			62	TTT	+	trna
tRNA-Leu (CUN)	*L2*			55	TAA	+	trna
tRNA-Ser (AGY)	*S1*			52	TCT	+	trna
NADH dehydrogenase subunit 2	*nad2*	GTG	T	844		+	protein
tRNA-Ile	*I*			60	GAT	+	trna
tRNA-Arg	*R*			55	ACG	+	trna
tRNA-Gln	*Q*			55	TTG	+	trna
tRNA-Phe	*F*			61	GAA	+	trna
Cytochrome b	*cytB*	GTG	TAG	1107		+	protein
tRNA-Leu (UUR)	*L1*			57	TAG	+	trna
Cytochrome c oxidase subunit 3	*cox3*	GTG	TAG	768		+	protein
tRNA-Thr	*T*			55	TGT	+	trna
NADH dehydrogenase subunit 4	*nad4*	TTG	TAG	1230		+	protein
Cytochrome c oxidase subunit 1	*cox1*	TTG	TAG	1578		+	protein
tRNA-Cys	*C*			56	GCA	+	trna
tRNA-Met	*M*			60	CAT	+	trna
tRNA-Asp	*D*			59	GTC	+	trna
tRNA-Gly	*G*			56	TCC	+	trna
Cytochrome c oxidase subunit 2	*cox2*	TTG	TAG	699		+	protein
tRNA-His	*H*			56	GTG	+	trna
Large ribosomal RNA (16 S rRNA)	*rrnL*			963		+	ribosome, large
NADH dehydrogenase subunit 3	*nad3*	TTG	TAA	354		+	protein
NADH dehydrogenase subunit 5	*nad5*	ATT	T	1585		+	protein
tRNA-Ala	*A*			56	TGC	+	trna
tRNA-Pro	*P*			56	TGG	+	trna
tRNA-Val	*V*			57	TAC	+	trna
NADH dehydrogenase subunit 6	*nad6*	TTG	TAG	435		+	protein

**Fig. 2:**
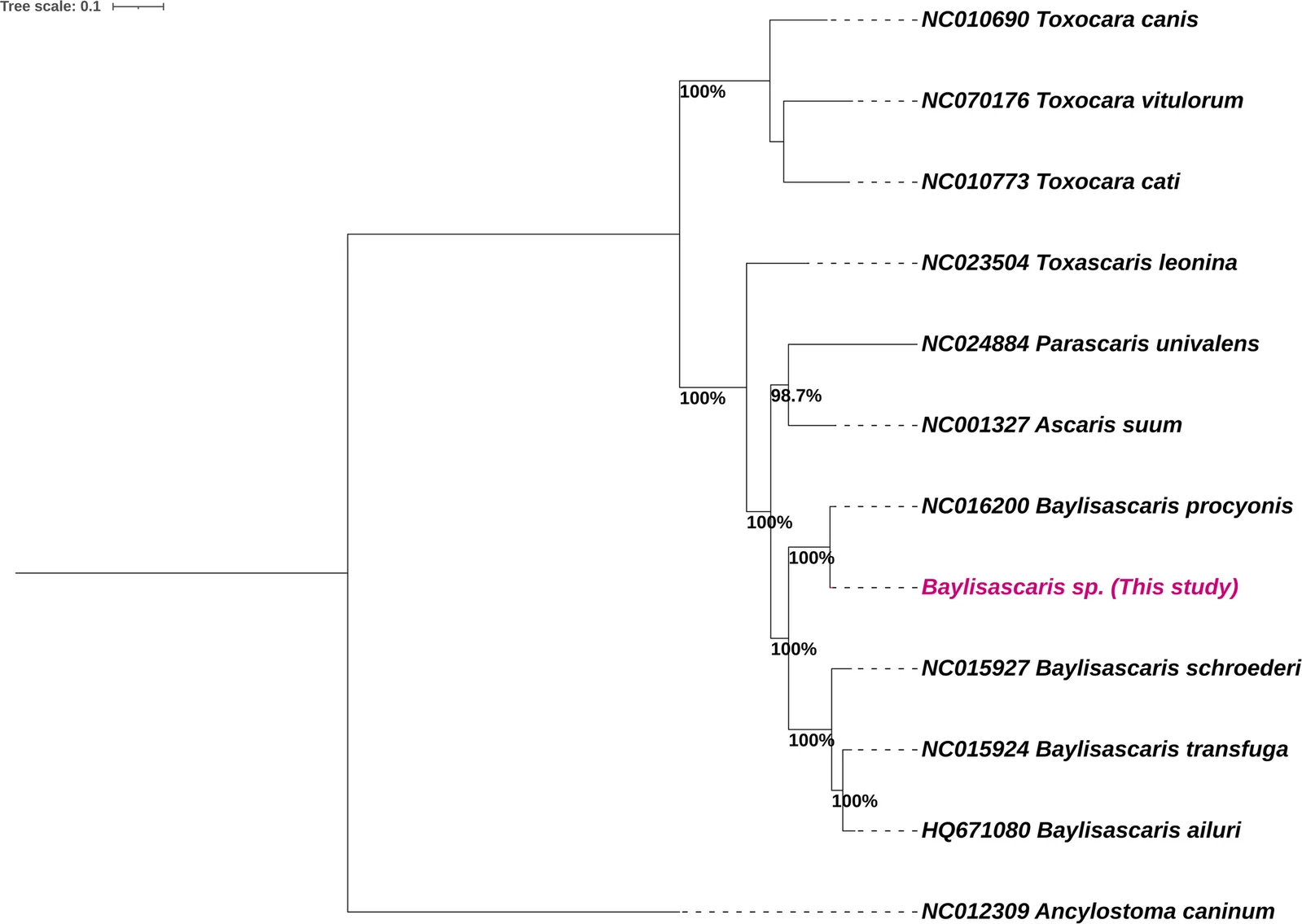
The phylogenetic relationship of *Baylisascaris* sp. from a skunk with ten other members of the family Ascarididae. A maximum-likelihood (ML) phylogenetic tree was constructed in IQTREE based on concatenated sequences of 14 mitochondrial genes (12 protein-coding genes and 2 rRNA genes). Bootstrap ML support values are shown at nodes. The tree is rooted with sequences from *Ancylostoma caninum* (accession no. NC_012309)

Phylogenetic analysis of the 14 concatenated mitochondrial genes (12 protein-coding genes and 2 rRNA genes) is presented in Fig. 2. The skunk-derived *Baylisascaris* from this study is in Clade 1, grouping with *B. procyonis*, forming a strongly supported monophyletic clade (100% bootstrap support). Clade 2 is comprised of *B. ailuri*, *B. schroederi*, and *B. transfuga*. Other *Baylisascaris* spp. listed in Sprent (1968) and those analyzed in previous molecular studies (Tokiwa et al. 2014; Pérez Mata et al. 2016; Camp et al. 2018; Barrera et al. 2022), including *B. devosi* from a pine marten, *B. potosis* from kinkajous, *B. tasmaniensis* from a Tasmanian devil, *B. laevis* from a marmot, and *B. venezuelensis* from spectacled bears, were not analyzed here due to lack of complete mitochondrial genome data for these species. Our findings further support the growing evidence that skunk-derived and raccoon-derived *Baylisascaris* are closely related with low levels of genetic differentiation, reinforcing the need to obtain additional data for a taxonomic assessment of *Baylisascaris* from these hosts (Camp et al. 2018; Gu et al. 2024).

Clarifying the taxonomic relationship between skunk- and raccoon-derived *Baylisascaris* species has direct implications for public health. Raccoons and skunks exhibit overlapping geographic ranges throughout much of the United States. If skunks are infected with *B. procyonis*, or if skunk-associated *Baylisascaris* and *B. procyonis* represent the same species, skunks, in addition to raccoons, would contribute to environmental contamination, and the zoonotic risk would be equivalent. However, even if they are distinct species, skunk-derived *Baylisascaris* species remain a public health concern given the capacity for larval tissue migration in humans (Kazacos 2001). The epidemiological significance of *Baylisascaris* spp. from skunks requires further investigation.

## Supplementary Information

Below is the link to the electronic supplementary material.

Supplementary figure 1 Supplemental Fig. 1. Morphological features of an immature Baylisascaris sp. recovered from a skunk. (**A**) Anterior end showing the three characteristic lips typical of members of the family Ascarididae. (**B**) Same as (**A**) with lips outlined for clarity. (**C**) Posterior end of the specimen (PNG 4.20 MB)

High Resolution Image (TIF 4.39 MB)

## Data Availability

The dataset generated and analyzed during the current study is available via the National Center for Biotechnology Information (NCBI; www.ncbi.nlm.nih.gov) GenBank database under accession number PZ025724, with raw sequencing data deposited in the NCBI sequence read archive (SRA) database under accession number SAMN61218381 (BioProject: PRJNA1484093).

## References

[CR1] Barrera MA, Janes JK, Gorrell JC (2022) Molecular phylogenetics and systematics of two enteric helminth parasites (*Baylisascaris laevis* and *Diandrya vancouverensis*) in the Vancouver Island marmot (*Marmota vancouverensis*). Int J Parasitol Parasites Wildl 19:301–310. 10.1016/j.ijppaw.2022.11.006 PMC9691466 36438195

[CR2] Camp LE, Radke MR, Shihabi DM, Pagan C, Yang G, Nadler SA (2018) Molecular phylogenetics and species-level systematics of *Baylisascaris*. Int J Parasitol Parasites Wildl 7(3):450–462. 10.1016/j.ijppaw.2018.09.010 PMC6275171 30568876

[CR3] Capella-Gutiérrez S, Silla-Martínez JM, Gabaldón T (2009) trimAl: a tool for automated alignment trimming in large-scale phylogenetic analyses. Bioinformatics 25(15):1972–1973. 10.1093/bioinformatics/btp348 PMC2712344 19505945

[CR4] Choi Y, Mason S, Ahlborn M, Zscheile B, Wilson E (2017) Partial molecular characterization of the mitochondrial genome of *Baylisascaris columnaris* and prevalence of infection in a wild population of Striped skunks. Int J Parasitol Parasites Wildl 6(2):70–75. 10.1016/j.ijppaw.2017.03.009 PMC5403792 28462088

[CR5] de Sena Brandine G, Smith AD (2021) Falco: high-speed FastQC emulation for quality control of sequencing data. F1000Research 8:1874. 10.12688/f1000research.21142.2 PMC7845152 33552473

[CR6] Dierckxsens N, Mardulyn P, Smits G (2016) NOVOPlasty: de novo assembly of organelle genomes from whole genome data. Nucleic Acids Res 45(4):e18–e18. 10.1093/nar/gkw955 PMC5389512 28204566

[CR7] Franssen F, Xie K, Sprong H, van der Giessen J (2013) Molecular analysis of *Baylisascaris columnaris* revealed mitochondrial and nuclear polymorphisms. Parasit Vectors 6(1):124. 10.1186/1756-3305-6-124 PMC3643864 23627901

[CR8] Graeff-Teixeira C, Morassutti AL, Kazacos KR (2016) Update on baylisascariasis, a highly pathogenic zoonotic infection. Clin Microbiol Rev 29(2):375–399. 10.1128/cmr.00044-15 PMC4786883 26960940

[CR9] Grant JR, Enns E, Marinier E, Mandal A, Herman EK, Chen C-y, Van Domselaar GM, Stothard G P (2023) Proksee: in-depth characterization and visualization of bacterial genomes. Nucleic Acids Res 51(W1):W484–W492. 10.1093/nar/gkad326 PMC10320063 37140037

[CR10] Gu X-H, Chen H-X, Hu J-J, Li L (2024) Morphology and ASAP analysis of the important zoonotic nematode parasite *Baylisascaris procyonis* (Stefahski and Zarnowski, 1951), with molecular phylogenetic relationships of *Baylisascaris* species (Nematoda: Ascaridida). Parasitology 151(2):200–212. 10.1017/S0031182023001312 PMC10941036 38087962

[CR11] Havird JC, Santos SR (2014) Performance of single and concatenated sets of mitochondrial genes at inferring metazoan relationships relative to full mitogenome data. PLoS ONE 9(1):e84080. 10.1371/journal.pone.0084080 PMC3891902 24454717

[CR12] Jex AR, Waeschenbach A, Littlewood DTJ, Hu M, Gasser RB (2008) The Mitochondrial Genome of *Toxocara canis*. PLoS Negl Trop Dis 2(8):e273. 10.1371/journal.pntd.0000273 PMC2483351 18682828

[CR13] Kalyaanamoorthy S, Minh BQ, Wong TKF, von Haeseler A, Jermiin LS (2017) ModelFinder: fast model selection for accurate phylogenetic estimates. Nat Methods 14(6):587–589. 10.1038/nmeth.4285 PMC5453245 28481363

[CR14] Katoh K, Misawa K, Kuma Ki, Miyata T (2002) MAFFT: a novel method for rapid multiple sequence alignment based on fast Fourier transform. Nucleic Acids Res 30(14):3059–3066. 10.1093/nar/gkf436 PMC135756 12136088

[CR15] Kazacos KR (2001) *Baylisascaris procyonis* and related species. In: Samuel WM, Pybus MJ, Kocan AA (eds) Parasitic diseases of wild mammals, 2nd edn. Iowa State University, Ames, IA, pp 301–341. 10.1002/9780470377000.ch11

[CR16] Krueger F, James F, Ewels P, Afyounian E, Weinstein M, Schuster-Boeckler B, sclamons Hulselmans G (2023) FelixKrueger/TrimGalore: v0.6.10 -add default decompression path. 10.5281/zenodo.7598955

[CR17] Laslett D, Canback B (2004) ARAGORN, a program to detect tRNA genes and tmRNA genes in nucleotide sequences. Nucleic Acids Res 32(1):11–16. 10.1093/nar/gkh152 PMC373265 14704338

[CR18] Letunic I, Bork P (2021) Interactive Tree Of Life (iTOL) v5: an online tool for phylogenetic tree display and annotation. Nucleic Acids Res 49(W1):W293–W296. 10.1093/nar/gkab301 PMC8265157 33885785

[CR19] Nguyen L-T, Schmidt HA, von Haeseler A, Minh BQ (2014) IQ-TREE: a fast and effective stochastic algorithm for estimating maximum-likelihood phylogenies. Mol Biol Evol 32(1):268–274. 10.1093/molbev/msu300 PMC4271533 25371430

[CR20] Okimoto R, Macfarlane JL, Clary DO, Wolstenholme DR (1992) The mitochondrial genomes of two nematodes, *Caenorhabditis elegans* and *Ascaris suum*. Genetics 130(3):471–498. 10.1093/genetics/130.3.471 PMC1204866 1551572

[CR21] Pérez Mata A, García Pérez H, Gauta Parra J (2016) Morphological and molecular description of *Baylisascaris venezuelensis*, n. sp. from a natural infection in the South American spectacled bear *Tremarctos ornatus* Cuvier, 1825 in Venezuela. Neotrop Helminthol 10:85–103. 10.24039/rnh2016101732

[CR22] Sapp SGH, Gupta P, Martin MK, Murray MH, Niedringhaus KD, Pfaff MA, Yabsley MJ (2017) Beyond the raccoon roundworm: the natural history of non-raccoon *Baylisascaris* species in the New World. Int J Parasitol Parasites Wildl 6(2):85–99. 10.1016/j.ijppaw.2017.04.003 PMC5429227 28529879

[CR23] Sprent JFA (1968) Notes on *Ascaris* and *Toxascaris*, with a definition of *Baylisascaris* gen. nov Parasitol 58(1):185–198. 10.1017/S0031182000073534 4868445

[CR24] Tokiwa T, Nakamura S, Taira K, Une Y (2014) *Baylisascaris potosis* n. sp., a new ascarid nematode isolated from captive kinkajou, *Potos flavus*, from the Cooperative Republic of Guyana. Parasitol Int 63(4):591–596. 10.1016/j.parint.2014.03.003 24662055

[CR25] Trifinopoulos J, Nguyen L-T, von Haeseler A, Minh BQ (2016) W-IQ-TREE: a fast online phylogenetic tool for maximum likelihood analysis. Nucleic Acids Res 44(W1):W232–W235. 10.1093/nar/gkw256 PMC4987875 27084950

[CR26] Xie Y, Zhang Z, Niu L, Wang Q, Wang C, Lan J, Deng J, Fu Y, Nie H, Yan N, Yang D, Hao G, Gu X, Wang S, Peng X, Yang G (2011) The Mitochondrial Genome of *Baylisascaris procyonis*. PLoS ONE 6(10):e27066. 10.1371/journal.pone.0027066 PMC3203944 22046447

[CR27] Zhang J (2024) Phylotools: phylogenetic tools for eco-phylogenetics, version 0.2.5. GitHub. https://github.com/helixcn/phylotools

[CR28] Zhang L, Sun K, Csorba G, Hughes AC, Jin L, Xiao Y, Feng J (2021) Complete mitochondrial genomes reveal robust phylogenetic signals and evidence of positive selection in horseshoe bats. BMC Ecol Evol 21(1):199. 10.1186/s12862-021-01926-2 PMC8565063 34732135

